# Glaucoma in India: Current status and the road ahead

**DOI:** 10.4103/0301-4738.73678

**Published:** 2011-01

**Authors:** Ravi Thomas

**Affiliations:** Queensland Eye Institute, University of Queensland, Brisbane, Australia. E-mail: ravi.thomas@qei.org.au

It is easy for the informed insider to be pessimistic about glaucoma in India. While we were concentrating on cataract surgery, glaucoma snuck up on us.[1,2] There are now an estimated 12 million people affected by glaucoma in India, the majority of whom are undiagnosed.[3] By 2020, this is expected to be 16 million.[4] How are we dealing with this and what should our national policy for the future be?

The major element of any glaucoma strategy must be “case detection”: When patients present to us for any reason, we should use that “opportunity” to detect glaucoma.[5] The goal should be to at least detect and manage the “in our face” glaucomas, those clear-cut cases with established functional loss which remain undiagnosed, primarily because a comprehensive examination (slit lamp, intraocular pressure, gonioscopy, dilated disk examination) has not been performed. The usual excuses for incomplete examination include the standard “poor developing country” groan along with the “cost of slit lamps, applanation tonometers and diagnostic lenses” moan. This is not unusual even in the environs of an otherwise well-appointed (furniture wise) out-patient clinic equipped with a spanking new Excimer laser facility. If finances are indeed an issue, shouldn’t we get our priorities right: bare professional necessities like applanations, slit lamps, and perimeters before Excimers?

Many do not perform such complete examinations on all new clinic patients because they are emulating their teachers and doing as they saw them do. Comprehensive eye examinations are routine in fewer than a handful of the 150 plus residency training programs, and automated perimeters, even if provided, are rarely used.[6] Large patient numbers and time constraints are genuine problems that need innovative strategies. Preliminary data suggest that a quick test with a frequency doubling perimeter (FDP) may help logistics by identifying those who need a full examination.[7]

As far as treatment strategy is concerned, the collaborative initial glaucoma treatment study (CIGTS) demonstrated that medical treatment is equivalent to surgical treatment.[8] Stand this on its head to read that surgical treatment is equivalent to medical treatment: One could argue that if we had to fund a glaucoma management initiative in India, it would have to be initial surgery, provided the diagnosis is made in a modern manner and surgery is performed by adequately trained ophthalmologists. Considering the potential iatrogenic complications, we should insist on the demonstration of a functional defect in any patient being considered for incisional surgery. While an inexpensive Bjerrum’s screen is certainly sufficient for this purpose, the screening mode of the FDP is more likely to be used routinely. Of course the other major problem is that glaucoma surgery is not taught routinely in residency programs.[6] Also, unlike cataract surgery, glaucoma management (including surgery) cannot be taught in a quick 1 month course.

Glaucoma fellowships around the country are a step in the right direction. However, those joining such programs are usually graduates of suboptimal residency training. At the L.V. Prasad Eye institute, we were forced to set aside >3 months of the fellowship to teach basic ophthalmic skills that should have been mastered during residency. These constraints compromise the research component in such fellowships. I know of only one residency and one fellowship program that formally teaches research methodology. The basic training issue also has implications for interpretation of research data. How valid and reliable are findings that have been obtained by someone trained for only 2 weeks in techniques (like gonioscopy) that they may have never used prior to recruitment for a specific research project?

The good news is that we are beginning to think about diseases other than cataracts. The World Health Organization (WHO) recently convened an expert panel to discuss approaches to glaucoma in developing countries. The consensus was that glaucoma cannot be approached in isolation; any strategy must be integrated into an overall approach to blindness. Also, the impact of a glaucoma program cannot be measured in terms of raw surgical numbers.

Glaucoma has to be managed by all ophthalmologists, leaving subspecialists to handle the more difficult cases. In the short term, we need a critical mass of trainers: The trainer of trainers type approach modified to cater to the skills required for glaucoma. But all such short-term training programs, including current fellowships, do not address the more basic issue. If we are serious about prevention of blindness, including that from glaucoma, then residency training programs must improve. Residency programs must teach modern clinical and surgical ophthalmic skills including those required to manage glaucoma.[6]

Achieving all this requires more than just political will; any change has to start with us. Edicts from a ministry accompanied by the usual workshops and short-term training programs are not enough. We must acknowledge that we have a problem and recognize that throwing money and equipment alone at it does not work.[6] We must also recognize that glaucoma is unlike cataract, and trying to measure outcomes purely by surgical numbers can have severe consequences.

India led the way by developing the first national program for the prevention of blindness. We now have an opportunity to create a model integrating glaucoma (and other specialized care). Let us bring the best ophthalmology and glaucoma talent together with the best public health brains to develop a quality-oriented long-term plan using appropriate outcome measures. Improve residency programs and thereby form a base that provides well-trained ophthalmologists. Use managers with an established track record for implementation and monitoring. Include the teaching and execution of operational research to answer clinically relevant questions such as the role of primary laser. Also, build in accountability at all levels, starting at the top.

This editorial has been as difficult to write as it will be for some of my peers to accept. My suggestions are made in all sincerity, knowing that success demands a concerted sincere effort. I hope that those in a position to make a difference will consider the truth of what I say, and take the actions which must be taken. And friends, please do not shoot the messenger.
